# Microwave synthesised Pd–TiO_2_ for photocatalytic ammonia production†

**DOI:** 10.1039/c8ra09762c

**Published:** 2019-02-22

**Authors:** Jake M. Walls, Jagdeep S. Sagu, K. G. Upul Wijayantha

**Affiliations:** Energy Research Laboratory, Department of Chemistry, Loughborough University Loughborough LE11 3TU UK j.m.walls2@lboro.ac.uk

## Abstract

Palladium doped anatase TiO_2_ nanoparticles were synthesised by a rapid (3 min) one-pot microwave synthesis technique at low temperature and pressure. After being fully characterised by SEM, XRD, Raman, XPS and EDX, photocatalytic nitrate reduction and ammonia production were studied over various dopant levels between 0–3.97 wt% Pd and compared to similar previous literature. Improved yields of ammonia were observed with most dopant levels when compared to non-doped microwave synthesised TiO_2_ with 2.65 wt% found to be the optimum dopant level producing 21.2 μmol NH_3_. Electrochemical impedance spectroscopy of TiO_2_ and Pd–TiO_2_ photoelectrodes revealed improvements in charge transfer characteristics at high Pd dopant levels.

## Abstract 2

A rapid 3 minute one-pot microwave synthesis of Pd–TiO_2_ showing improved activity for photocatalytic nitrate reduction into ammonia.

## Graphical abstract

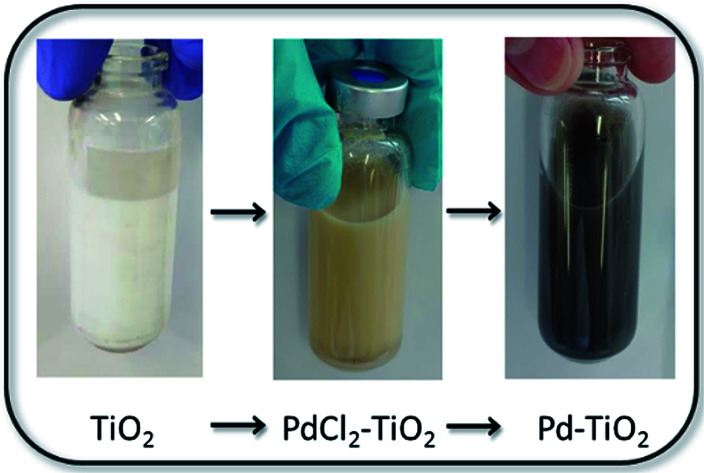

## 1. Introduction

Ammonia (NH_3_) is the most produced commercial chemical in the world with its production ever increasing, as in 2012 it was up to 160 million tons per annum.^1^ Its significant use is in the fertilizer industry where over-manuring has caused a significant increase of the concentration of harmful nitrate ions (NO_3_^−^) in groundwater.^2^ Nitrate ions and their derivatives are considered water pollutants as toxic levels have been shown to cause methemoglobinemia in infant children and act as a possible promotor of carcinogenesis.^3,4^ Release of nitrate into groundwater is well-known to cause eutrophication destroying ecosystems with higher levels of nitrate. This has led to considerable research into nitrate reduction *via* a range of different techniques including biological,^5^ electrochemical,^6–8^ and catalytic methods.^9–11^ Among these methods photocatalytic reduction stands out as a sustainable, potentially scalable and environmentally friendly solution with the ability to reduce aqueous nitrate to nitrogen or ammonia utilising only a suitable photocatalyst, water and sunlight. Typical photocatalysts are low cost and non-toxic, enabling ammonia production without the need of high energy input as in Haber–Bosch processes (*i.e.* high temperatures and pressures). This drastically reduces levels of CO_2_ released into the atmosphere when the photocatalytic method is compared to Haber–Bosch process as well as other alternative methods.

A number of photocatalytic materials both doped and undoped; such as TiO_2_,^2,12–17^ ZnO,^18–20^ SrTiO_3_,^21^ CdS,^22–24^ ZnS,^25,26^ Fe_2_O_3_,^19,27^ and ZrO_2_(ref. 19) have been studied for photocatalytic nitrate reduction to date. Among them titanium dioxide (TiO_2_) has been the most popular due to its good photocatalytic activity, stability within a wide pH window and non-toxicity in nature.^28,29^ Due to its large band-gap, the research focus to date has been to improve its light absorption characteristics by doping without compromising the photocatalytic performance.^11,30–32^ Palladium has been one of the common dopants which has been investigated for nitrate reduction due to its well-known catalytic properties and hydrogen adsorption capabilities,^33,34^ and has been shown previously to have photocatalytic nitrate reduction properties.^2,31,35,36^

Microwave synthesis is an established technique today. Microwave heat generation in materials is fundamentally different to that of conventional heating methods such as radiant, conduction and convection heating. Conventional heating uses heated elements to transfer heat to the reaction vessel as a heat transfer process, whereas microwave irradiation is an energy conversion process where the heat is generated within the reaction mixture itself. Therefore, inorganic materials can be produced with unique properties using microwave synthesis that cannot be accomplished by other conventional thermal synthesis methods.^37^ It has been reported that microwave synthesis of materials, including photocatalysts, can have many advantages including high efficiency, rapid synthesis capability which significantly improves mono-dispersity, controllability of morphology and high catalytic activity.^37–40^ In contrast, conventional methods for photocatalyst production typically involve lengthy heating procedures and often can lead to non-uniform crystallinity and dopant distribution, which reflect the non-uniform thermal distribution at microscopic scale.^38,40^ Although improved photocatalytic activity of microwave synthesised photocatalysts has been studied previously,^39^ none have been applied to ammonia production *via* photocatalytic nitrate reduction.

Herein, we present the rapid microwave synthesis method of Pd–TiO_2_ and its ability to conduct photocatalytic ammonia production. This is the first report where ammonia production *via* photocatalytic nitrate reduction is completed using microwave synthesised photocatalysts. In this study, we investigate the photocatalytic nitrate reduction properties microwave synthesised TiO_2_ and various dopant levels of Pd–TiO_2_ in comparison to previously reported literature methods of similar photocatalysts with conventional synthesis techniques. In addition, photocurrent and EIS studies of microwave synthesised TiO_2_ and Pd–TiO_2_ photoelectrodes were studied. These findings are particularly important in the context of current efforts to find a more sustainable means of sustainable ammonia production as well as reduction of nitrates in wastewater.

## 2. Experimental

### 2.1. Microwave synthesis of Pd–TiO_2_

Titanium(iv) isopropoxide (12.9 mmol, Sigma Aldrich 99.999% purity) and palladium(ii) chloride (0.4 mmol, Sigma Aldrich, 99.999% trace metals purity) were fully dissolved in 20 mL of deionised water. The amount of palladium was varied for different dopant levels of Pd–TiO_2_. This solution is vigorously stirred, before being placed in a 20 mL quartz microwave reaction vessel with a magnetic stirrer. In a typical reaction, the reaction vessel was held at 150 °C and ∼11 bar overpressure for 3 minutes utilising a Biotage Initiator EXP 8 Reactor (Fig. S1†). The nanopowder suspension changes colour from white to cream when the titanium isopropoxide and palladium chloride are added respectively, and then cream to black before and after the microwave reaction respectively (Fig. S2†).^41^ Where no titanium isopropoxide is present, palladium(ii) chloride is reduced in to Pd/PdO.

### 2.2. Characterisation of Pd–TiO_2_ photocatalyst

Powders were characterised by a Bruker D8 Advance X-ray diffractometer (XRD) with monochromatic Cu Kα (*λ* = 1.54 Å) in reflection geometry using a Lynxeye PSD detector. Reflections were observed over a 2*θ* range of 10–80° using a step size of 0.0039° and a time per step of 2.1 s. The phase and crystallinity of produced powders were then analysed and characterised in comparison to references from the inorganic crystal structure database (ICSD) for known reflections for each material and phase. The surface composition of photocatalytic powders was analysed using XPS analysis to reveal percentage levels of dopant on the surface of TiO_2_ powders. Measurements were conducted with a thermo scientific spectrophotometer (model K-α) over a 400 μm^2^ area. Raman spectra were measured using a HORIBA Jobin Yvon LabRAM HR (632.8 nm He–Ne laser) Raman spectrometer across the wavenumber range of 100–2000 cm^−1^. A Leo 1530VP field emission gun (FEG)-SEM was used to examine the surface morphology at an accelerating voltage of 5 kV and a working distance of 5 mm. EDX spectroscopy was also carried out to determine the bulk Pd/Ti ratio to compare to XPS surface ratio.

### 2.3. Photocatalytic studies

Photocatalytic reactions were conducted in a 1000 ml photochemical reactor (Lelesil Innovative Systems, Fig. S3†) with a 400 W UV lamp. In a typical photocatalytic experiment 500 mg of photocatalyst was suspended in 400 ml of deionised water and stirred for 3 hours under irradiation and a flow of nitrogen (500 ml min^−1^). Each powder was additionally tested for photocatalytic nitrogen reduction, but no ammonia was detected without the presence of nitrate ions in solution. For each reaction 121 ppm of KNO_3_ was added as the nitrogen source and the system was stirred at 600 rpm from the addition of the nitrate until the end of reaction. The system was flushed with nitrogen for 30 minutes before the reaction start and held throughout the reaction at a flow of 350 ml min^−1^. Temperature was controlled to be 25 °C ± 5 °C for each reaction. Samples of 5 mL, were taken before irradiation and every 30 minutes until completion and tested for ammonia.

### 2.4. Ammonia detection

Ammonia was detected *via* the Berthelot colorimetric method reported by Grayer *et al.*^42,43^ This modified Berthelot reaction utilised two individual reagents that when mixed in a solution containing NH_3_ (1–400 μM) would change the colour of solution from to produce the dye molecule indophenol blue.^44^ Phenol reagent: phenol (3.0 g, Sigma Aldrich, 98%) and sodium nitroferricyanide (0.015 g, Sigma Aldrich, ≥99%) were dissolved in 50 ml deionised water and stored at 3 °C. Sodium hypochlorite reagent: sodium hydroxide (1.5 g, Sigma Aldrich, ≥97%) and sodium hypochlorite solution (2.4 ml, Sigma Aldrich, >8% active chlorine) were dissolved in 50 ml deionised water and stored at 3 °C. Procedure: 0.5 ml of both reagents were added to 2 ml of diluted sample and compared to calibration standards *via* the same method. Spectrophotometric measurements were conducted across wavelengths 400–800 nm on a Lambda 35 Perkin Elmer UV-Vis spectrophotometer and absorbance analysed at 630 nm specifically.

### 2.5. Electrochemical studies

Photocatalyst powders were ultrasonically dispersed in ethanol (150 mg, 150 ml) and drop cast onto roughly 2 × 1 cm Fluorine-doped Tin Oxide (FTO), for each electrode 3 ml of dispersion was added dropwise and ethanol allowed to evaporate. Electrochemical measurements were conducted on an Eco Chemie Autolab PGSTAT12 with a 150 W halogen lamp (Prior CL150) illumination source. The photoelectrodes were measured in a three-electrode mode configuration with Ag/AgCl 3 M KCl reference electrode and a platinum mesh counter electrode. The electrolyte was 0.2 M sodium sulphate (Sigma Aldrich, ACS reagent, 99.0%). A typical photocurrent response scan was held at 0.7 V *vs.* Ag/AgCl for a preconditioning step of 300 s before being measured for 240 s while illuminated every 20 s for 20 s. The electrochemical impedance spectroscopy (EIS) measurements were also conducted at 0.7 V *vs.* Ag/AgCl over a 5 mHz to 1 kHz frequency range and an amplitude of 0.01 V. The measured data was fitted and simulated using an equivalent circuit with two resistors, one in series and one in parallel, and a constant phase element in parallel on Nova 2.0 Software (Metrohm Autolab B.V.). All electrochemical measurements were conducted in an argon atmosphere, unless otherwise stated. Diffuse Absorbance measurements were carried out on the thin films using a Lambda 35 Perkin Elmer UV/Vis Spectrophotometer using a range of 330–800 nm at a scan rate of 420 nm min^−1^.

## 3. Results and discussion

### 3.1. SEM analysis

**Fig. 1:**
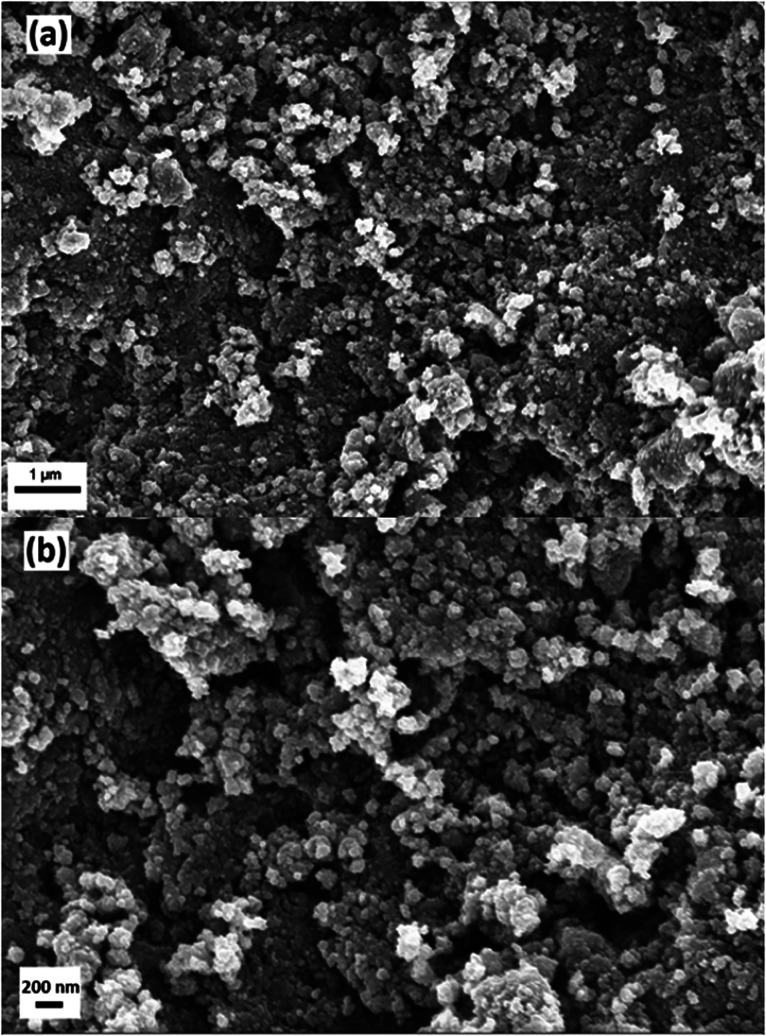
FEG-SEM images of microwave synthesised 3.97 wt% Pd–TiO_2_ powders at a magnification of (a) 25 000× and (b) 50 000×.

Doped and undoped Pd–TiO_2_ and TiO_2_ nanoparticles were synthesised using a microwave reactor for investigation of the photocatalytic activity for ammonia production *via* nitrate reduction. SEM studies show the formation of nanoparticles made *via* microwave synthesis. The SEM images were also utilised to determine the approximate particle size of the photocatalytic powders. Fig. 1 shows a typical SEM image of 3.97 wt% Pd–TiO_2_ powder at a couple of magnifications. Fig. 1b reveals that the approximate particle size is between 50–100 nm however it is observed that distinct particles can agglomerate more into larger particulates of 150–250 nm.

### 3.2. XRD analysis

**Fig. 2:**
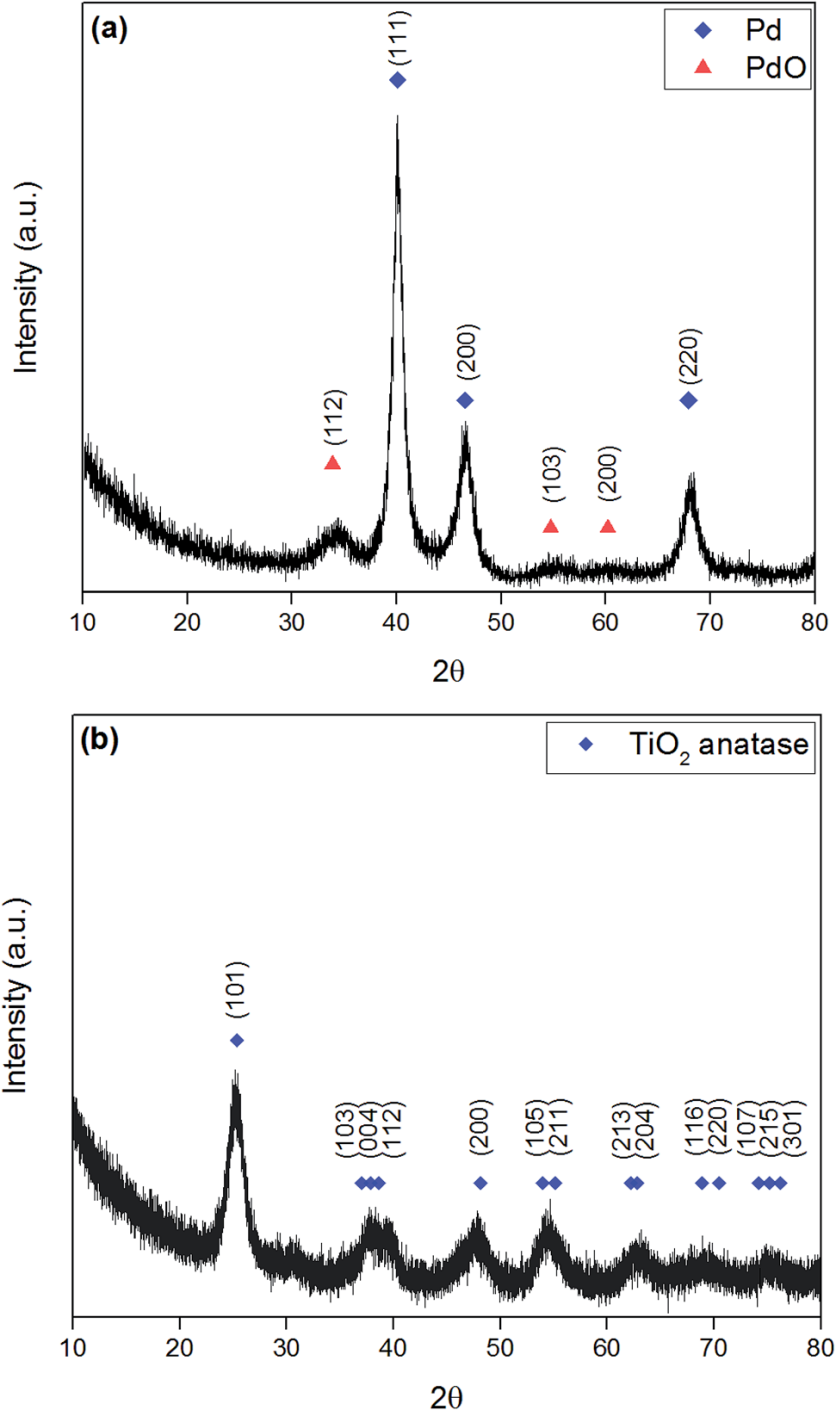
XRD pattern of microwave synthesised (a) Pd/PdO from PdCl_2_ reduction and (b) 3.97 wt% Pd–TiO_2_.

To observe whether we have formed the anatase crystalline phase and assess crystallinity of the photocatalysts, XRD studies were conducted both with and without the presence of the titanium precursor. This allows us to analyse the crystallinity of the photocatalyst as well as identifying all possible reflections that may be presented. Fig. 2a shows the XRD pattern of Pd/PdO nanopowder produced by reduction of PdCl_2_ precursor solution in a typical 3 minute microwave synthesis producing a mixed phase of Pd/PdO. Reflections are seen at 40.1°, 46.7° and 68.1° which correspond to the (111), (200) and (220) reflections of Pd, respectively (ICSD 00-046-1043). Furthermore, the presence of much weaker reflections at 33.9°, 54.8° and 60.2° correspond to reflections in (101), (112) and (103) planes of PdO (ICSD 00-043-1024), however these reflections are particularly low in intensity with relation to the noise. This correlates with XPS data revealing the majority phase present in Pd–TiO_2_ powders is the Pd^0^ metal with a small amount of PdO (see Fig. 4c). Fig. 2b displays a typical XRD pattern of microwave synthesised 3.97 wt% Pd–TiO_2_, revealing reflections between 20° and 80° which are corresponding to the anatase phase of TiO_2_ (ICSD 00-21-1272), with no evidence for the presence of rutile phase or Pd/PdO. The low intensity of the reflections is likely due to the rapid synthesis process of the powders as seen previously in literature.^45^ It is observed however that crystallinity slightly increases after microwave irradiation as shown by Fig. S4†, which reveals an XRD spectra of the beige coloured intermediate Pd–TiO_2_ before microwave irradiation. All similar reflections of anatase TiO_2_ (ICSD 00-21-1272) as previously shown were present, with no presence of Pd/PdO reflections.

### 3.3. Raman analysis

**Fig. 3:**
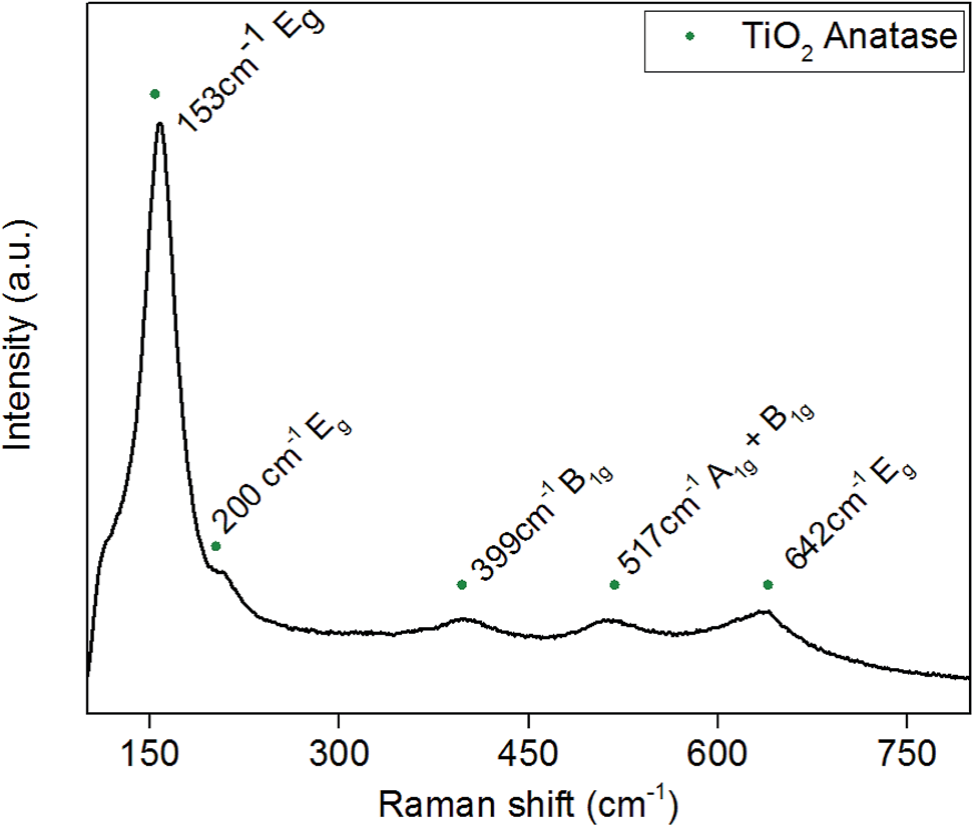
Typical Raman spectra of as-synthesised Pd–TiO_2_ powders.

Raman spectra were obtained to determine whether any amorphous phases of TiO_2_ or Pd were present in the powders, as there was no evidence of Pd in the XRD spectra. The Raman spectrum shown in Fig. 3 correlates with previous XRD data that anatase TiO_2_ is the majority phase present. Typical Raman spectra of anatase TiO_2_ has five active bonding modes at approximately 144 cm^−1^ (E_g_), 197 cm^−1^ (E_g_), 397 cm^−1^ (B_1g_), 518 cm^−1^ (A_1g_ + B_1g_) and 640 cm^−1^ (E_g_). Fig. 3 is the Raman spectrum of 3.97 wt% Pd–TiO_2_ which provides evidence for the presence of all the anatase active bonding modes stated above at around 153 cm^−1^, 200 cm^−1^, 399 cm^−1^, 517 cm^−1^ and 642 cm^−1^. In all spectra, the 1072 cm^−1^ peak for palladium is potentially indicated to be present with a very small peak, however, is too small to be separately identified from the noise. The incorporation of palladium has been shown in literature to cause small shifts in Raman active peaks which is seen in Fig. 3 with slight peak shifts compared to typical TiO_2_ bonding modes.^46,47^

### 3.4. XPS analysis

**Fig. 4:**
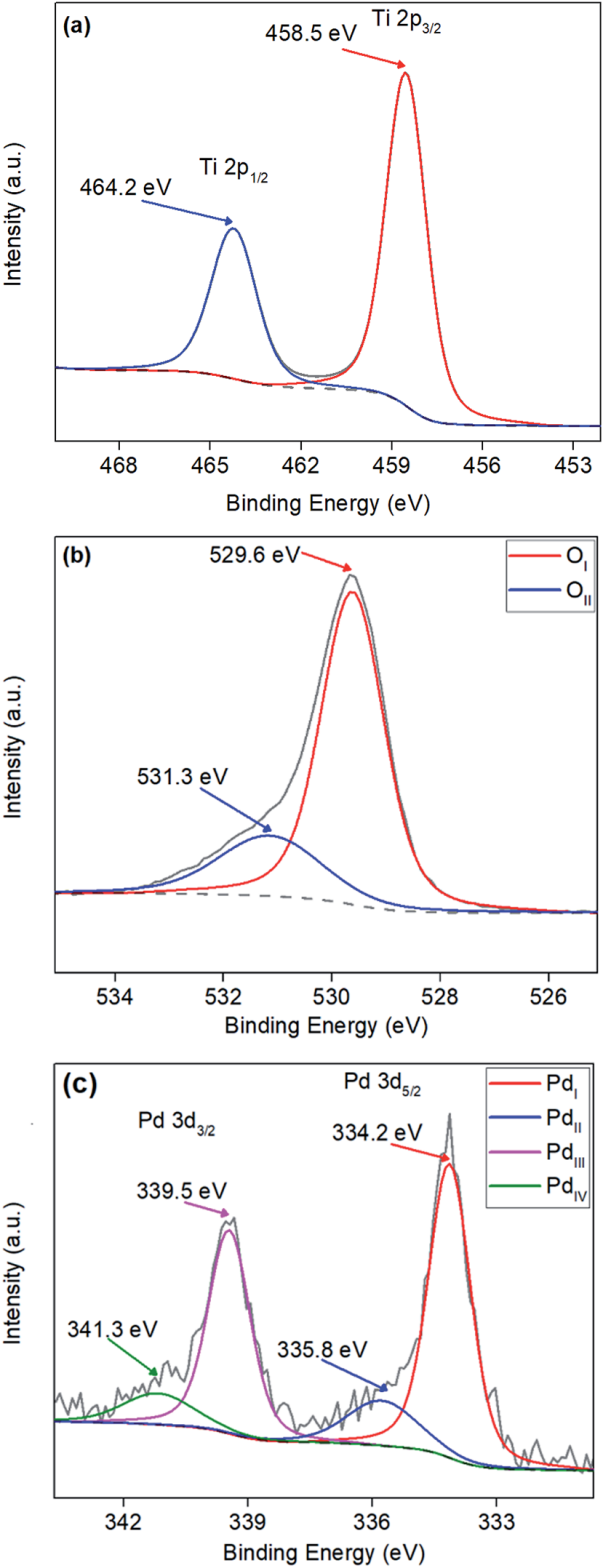
XPS analysis of (a) Ti 2p peaks, (b) O 1s peaks and (c) Pd 3d peaks of a typical microwave synthesised 3.97 wt% Pd–TiO_2_ photocatalyst.

XPS surface analysis was undertaken on TiO_2_ and Pd doped TiO_2_ powders to investigate the dopant level of Pd and the bonding modes of Pd, Ti and O. Powders varying from 0.27 wt% to 3.97 wt% Pd were analysed to determine whether the amount of Pd precursor utilised in the synthesis stage was still present in the doped TiO_2_ powders. Fig. 4a shows a sharp Gaussian peak at ∼458.5 eV corresponding to Ti 2p_3/2_ with a broader split spin orbit peak at Δ5.7 eV from the initial peak at ∼464.2 eV corresponding to Ti 2p_1/2_, both peaks can be attributed to the TiO_2_ structure as reported throughout literature.^48,49^ XPS peak values for Ti 2p_3/2_ and Ti 2p_1/2_ in all powders were within ±0.3 eV from the values shown in Fig. 4a. Fig. 4b illustrates the O 1s surface analysis of 3.97 wt% Pd–TiO_2_ revealing the typical O 1s peaks observed on all photocatalysts observed. The two peaks are denoted O_I_ and O_II_ relate to the O 1s and OH^−^ peaks respectively. The largest peak O_I_ at approximately 529.5 eV corresponds to the O^2−^ within the TiO_2_ anatase structure.^49^ The peak O_II_ shown at ∼531.3 eV correlates to the OH^−^ groups leftover on powders from the synthesis of TiO_2_ from titanium isopropoxide precursor, which follows a known reaction (see eqn (1)).^50–53^

1 Ti(OCH(CH_3_)_2_)_4_ + 2H_2_O → TiO_2_ + 4(CH_3_)_2_CHOH

Additionally, palladium was observed within the region of 330–345 eV with 2 well defined spin–orbit doublets of Pd 3d_5/2_ and Pd 3d_3/2_ (see Fig. 4c.). All spectra revealed Pd peaks with small oxide peaks present on the powders revealing a mixture between Pd^0^ and Pd^2+^ in the dopant, Pd^0^ has been indicated to be active towards nitrate reduction.^54^ In Fig. 4c, 4 peaks over the Pd region are denoted Pd_I_, Pd_II_, Pd_III_ and Pd_IV_. The Pd_I_ and Pd_II_ are two components of the Pd 3d_5/2_ peak located at ∼334.2 eV and ∼335.8 eV respectively, with Pd_I_ representing the Pd^0^ and Pd_II_ representing Pd^2+^. Table 1 shows quantitative data observed from XPS showing surface Pd/Ti ratio in comparison to bulk Pd/Ti ratio analysed *via* EDX of doped Pd–TiO_2_ powders.

**Table 1:** Pd/Ti ratios compared between synthesis, XPS surface analysis and EDX bulk analysis

Palladium dopant/wt%	Pd/Ti ratio added to synthesis/at%	XPS surface analysis, Pd/Ti ratio/at%	EDX bulk analysis, Pd/Ti ratio/at%
0.27	0.20	0.28^a^	0.14^a^
0.67	0.50	0.97	0.45
1.06	0.80	1.01	0.66
1.99	1.50	2.34	1.40
2.65	2.0	2.31	1.71
3.97	3.0	2.57	2.73

a Measured around the limit of detection.

### 3.5. EDX analysis

To determine where the palladium is present in the Pd–TiO_2_ nanopowder, XPS and EDX studies on the doped Pd–TiO_2_ were conducted and Pd/Ti ratio of both were compared to the Pd/Ti ratio added in the synthesis (see Table 1). Initially, we saw a larger Pd/Ti ratio on the surface than added into the synthesis from XPS analysis and a lower ratio in the bulk for all dopant levels other than 3.97 wt% Pd–TiO_2_. This indicates that the palladium present in the powder is mainly situated on the surface (first 10 nm). However, a higher Pd/Ti ratio is observed in the bulk EDX measurement when observing the 3.97 wt% Pd–TiO_2_ powder in comparison to the XPS, suggesting that at this point the surface is saturated with palladium and the rest is therefore observed in the bulk. Fig. S5† shows a typical 3.97 wt% EDX spectrum. The EDX analysis further confirms the presence of Pd throughout the structure of the photocatalysts.

### 3.6. Photocatalytic activity

**Fig. 5:**
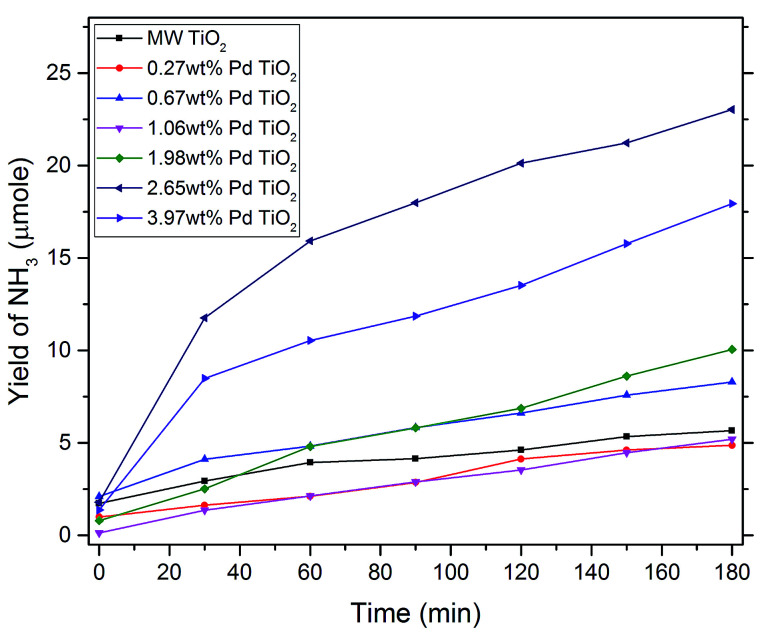
Yield of NH_3_ changing over reaction time of typical photocatalytic reaction for various dopant levels of microwave synthesised Pd–TiO_2_ (500 mg, 122 ppm NO_3_^−^, 3 h irradiation time, 400 W UV lamp).

Yields of ammonia for each dopant level were analysed and compared over photocatalytic reactions for 3 hours in Fig. 5. Microwave synthesised TiO_2_ (MW TiO_2_) was produced *via* the same synthesis just without the presence of the palladium precursor. Increases in yield were only observed after the dopant level was increased past 0.27 wt% with significant increases in yield in ammonia at with higher dopant levels of 2.65 wt% and 3.97 wt% Pd–TiO_2_, however the optimum dopant level was found to be 2.65 wt% out of all the photocatalytic powders tested. At dopant levels higher than 1.99 wt% we see most of increased Pd content being incorporated into the bulk of the material (see Table 1) and inherently increasing absorption characteristics of the photocatalysts (see Fig. S9†). Therefore, by improving light absorption characteristics of the photocatalysts, higher concentrations of e^−^/h^+^ pairs would be produced for use in photocatalytic nitrate reduction.

These experimental results concur with previous literature in that metal ion doping improves photocatalytic nitrate reduction ability of photocatalysts.^2,55^ In addition, it has been theorised that the metal ion dissociates the chemisorbed hydrogen to give rise to H_ads_ and thus reduce nitrate to ammonia with e^−^ and h^+^ pairs as shown in eqn (2) and (3) below.^11^ With increasing dopant concentration past 2.65 wt% yields begin to decrease with increasing palladium content, likely due to the higher recombination of e^−^/h^+^ pairs at very high dopant concentrations.^56,57^

2 NO_3_^−^ + 9H^+^ + 8e^−^ → NH_3_ + 3H_2_O

3 4H_2_O + 8h^+^ → 2O_2_ + 8H^+^

Table 2 compares the yield after reaction and shows how varying the dopant level affects the yield of NH_3_. The activity of photocatalyst powders were calculated normalising for time of reaction and amount of photocatalyst used to allow comparison to previous similar works. The activity of the as-synthesised microwave photocatalysts show vast increases in activity when compared to conventionally made powders when subjected to similar photocatalytic experimental conditions.^31,35^ However, some current studies have looked into co-doping, hole scavengers, higher nitrate concentration and smaller particles sizes to show improved yields.^2^ Ethanol and iso-propanol were investigated as hole scavengers in an attempt to improve ammonia yields, however this led to no significant yields of ammonia being observed. This could be due to increased selectivity towards nitrogen as the final reaction product instead of the ammonia in solution as seen in literature.^58^ All photocatalysts showed reproducible results with no observable degradation between photocatalytic reactions, revealing a reusability of recovered photocatalyst powder. As seen in Fig. S6,† where the same 2.65 wt% Pd–TiO_2_ photocatalyst powder underwent identical photocatalytic experiments with little variation in yield across the 4 repeated experiments.

**Table 2:** Photocatalytic ammonia production yields from various dopant level microwave synthesised Pd–TiO_2_ powders with comparison to previous literature work^31,35^

Author and year	Pd dopant level/wt%	Reported yield	Calculated activity^a^/μmol h^−1^ g^−1^	Lamp + power
Current study	0	3.9 μmol	26	UV 400 W
	0.27	3.9 μmol	2.6	UV 400 W
	0.67	6.2 μmol	4.1	UV 400 W
	1.06	5.1 μmol	3.4	UV 400 W
	1.98	9.3 μmol	6.2	UV 400 W
	2.65	21.2 μmol	14.2	UV 400 W
	3.97	16.6 μmol	11.0	UV 400 W
Kudo 1987^b^	0.3	0.2 μmol h^−1^	1.0	Xe 500 W
Ranjit 1997^b^	0.69	0.33 μmol	0.825	Xe 450 W

a Activity was calculated using experimental information given in previous literature.

b Only the best performing Pd–TiO_2_ powders were used in comparison.

### 3.7. Electrochemical analysis

**Fig. 6:**
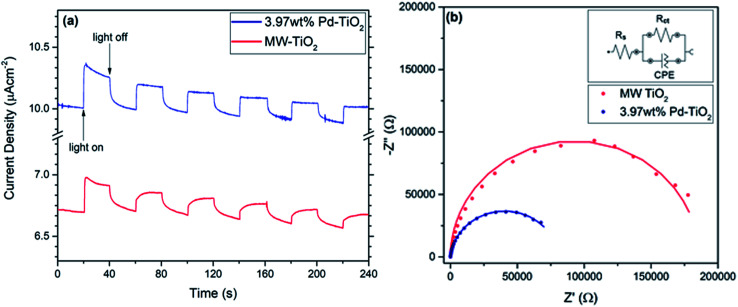
(a) Electrochemical photocurrent of MW-TiO_2_ and 3.97 wt% Pd–TiO_2_ photoelectrodes at 0.7 V *vs.* Ag/AgCl (b) Nyquist plot comparison for MW-TiO_2_ and 3.97 wt% Pd–TiO_2_ photoelectrodes, under 150 W halogen lamp illumination at 0.7 V *vs.* Ag/AgCl.

For electrochemical analysis of the photocatalysts, the efficiency of charge transfer and photocurrent response of both MW-TiO_2_ and 3.97 wt% Pd–TiO_2_ photoelectrodes were investigated. All measurements were conducted at 0.7 V *vs.* Ag/AgCl due to having the highest photocurrent response when held at this voltage compared to all others. It was assumed that the electrodes have a similar mass loading due to being produced *via* the exact same method, therefore it is possible to compare the performance of both MW-TiO_2_ and 3.97 wt% Pd–TiO_2_ photoelectrodes. As presented in Fig. 6a, a photocurrent response is seen, with 3.97 wt% Pd–TiO_2_ showing better performance starting with approximately 0.3 μA cm^−1^ and decaying to 0.2 μA cm^−1^ over the measurement. Meanwhile, MW-TiO_2_ starts with a response of approximately 0.2 μA cm^−1^ and decays to 0.1 μA cm^−1^. Due to the photostability of the photocatalyst shown earlier and the lack of evidence for palladium oxidation seen in cyclic voltammetry (see Fig. S6 and S7†), it is assumed that the mass loss, as a result of poor adhesion of the photocatalyst on the FTO glass substrate, is the cause of this degradation in photocurrent observed. Oxygen reduction appears to be observed when measurements were conducted in air at negative potentials but is not observed in an argon atmosphere.

Fig. 6b shows the Nyquist plots for both MW-TiO_2_ and 3.97 wt% Pd–TiO_2_ at 0.7 V *vs.* Ag/AgCl and 150 W halogen lamp illumination, similar to previous works in the field conducting EIS at a potential.^59–62^ The data was fitted and simulated with an equivalent circuit with components for the charge transfer resistance, *R*_ct_, solution resistance, *R*_s_ and a constant phase element for the semiconductor–electrolyte interface. The charge transfer resistance between the electrode–electrolyte interface can be calculated by the difference between both high and low frequency *x*-axis intercepts.^63,64^ Therefore as shown by 3.97 wt% Pd–TiO_2_ much smaller arc, it has a much smaller charge transfer resistance of 70 kΩ whereas, MW-TiO_2_ has a charge transfer resistance of 178 kΩ. This indicates that the doping with palladium reduces the charge transfer resistance and improves the activity of the catalyst, this could be a result of better conductivity or electron mobility, and thus correlates with higher photocatalytic activity noticed with higher dopant concentrations. Fig. S8† shows Nyquist plot comparisons between light and dark measurements for both MW-TiO_2_ and 3.97 wt% Pd–TiO_2_ photoelectrodes at 0.7 V *vs.* Ag/AgCl. Both showed smaller arcs in the light showing a decrease in charge transfer resistance while illuminated as expected, MW-TiO_2_ decreased from 195 kΩ to 178 kΩ where 3.97 wt% Pd–TiO_2_ decreased from 84 kΩ to 70 kΩ. Fig. S9† shows the diffuse absorbance measurements of the photoelectrodes revealing a typical strong UV absorption of TiO_2_ powders, however a large increase in baseline absorption is also observed as expected with Pd doping of TiO_2_ powders.^65^

## 4. Conclusions

In summary, we have reported a rapid one-pot microwave synthesis method to produce Pd–TiO_2_ anatase nanoparticles, utilising low temperature and pressure. The nanoparticles were then characterised *via* SEM, XRD and Raman revealing 50–100 nm particles with a few large agglomerates and an anatase majority phase powder. Surface analysis *via* XPS showed the presence of Pd^0^ and Pd^2+^ dopant in addition to TiO_2_ signature titanium and oxygen peaks. Photocatalytic nitrate reduction over various Pd dopant levels were conducted and yields of ammonia were compared to previous literature using similar photocatalysts revealing a vastly improved photocatalytic performance. Photoelectrodes were produced and analysed for electrochemical photocurrent and impedance spectroscopy revealing vast differences in charge transfer resistance with higher dopant concentrations. Finally, this is the first report of using microwave synthesised photocatalysts for use in photocatalytic ammonia production *via* nitrate reduction, with improved yields and a simple synthesis to be of significant interest to this field.

## Conflicts of interest

There are no conflicts of interest to declare.

## Supplementary Material

RA-009-C8RA09762C-s001
