# Supplementary material for: Microwave synthesised Pd–TiO2 for photocatalytic ammonia production
Source: RSC Adv. 2019 Feb 22;9(11):6387–94. doi: 10.1039/c8ra09762c (PMC9060955; doi:10.1039/c8ra09762c)
Supplement: RA-009-C8RA09762C-s001 [file RA-009-C8RA09762C-s001.pdf]

Electronic Supplementary Material (ESI) for RSC Advances.
This journal is © The Royal Society of Chemistry 2019

Supplementary Information

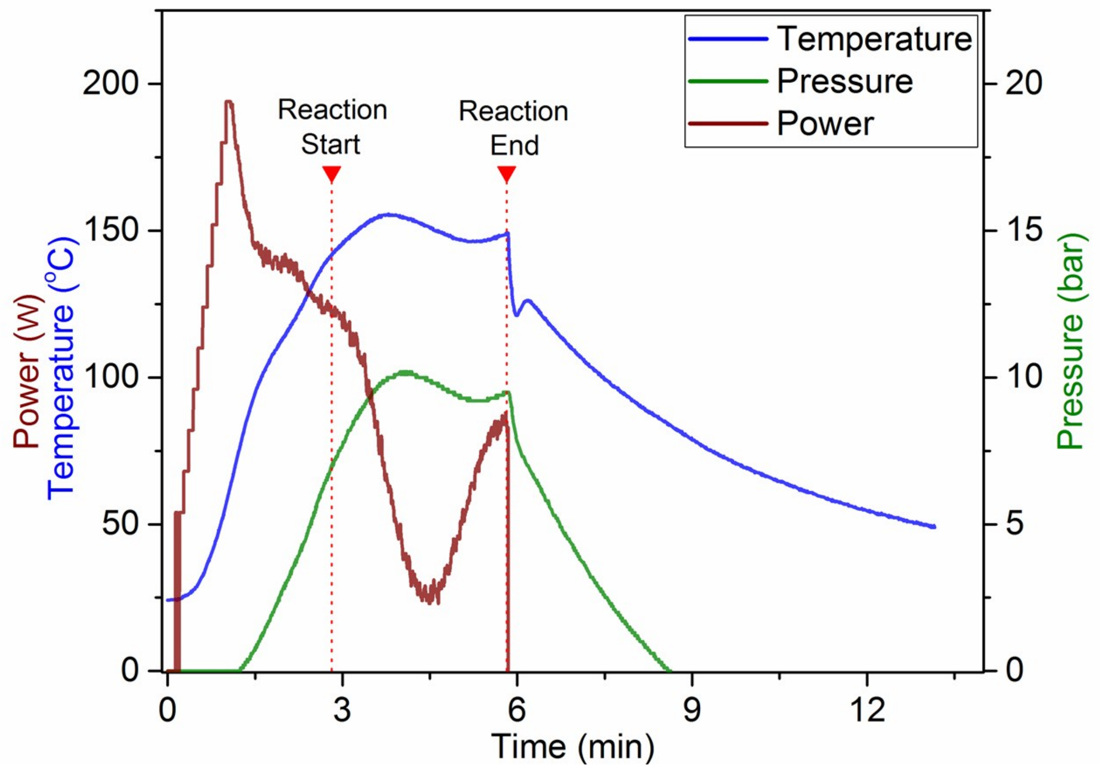

Figure S1. Graph showing experimental data of pressure, temperature and power during the microwave synthesis of 3.97wt% Pd-TiO_2

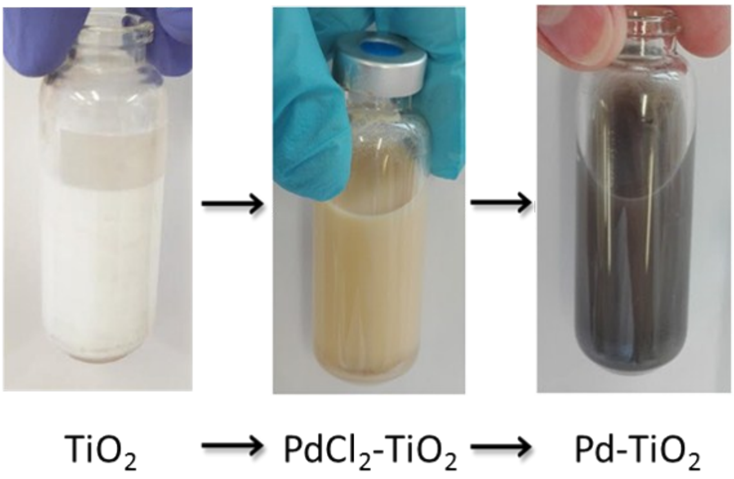

Figure S2. Pictures showing colour changes of nanopowder suspensions after the addition of Pd precursor and after the microwave synthesis.

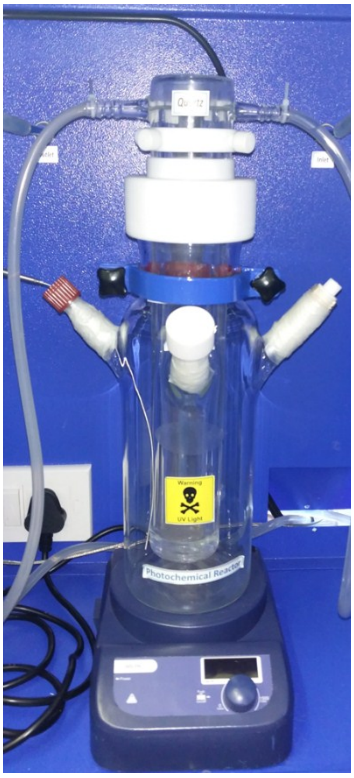

Figure S3. Picture of Lelesil Photocatalytic Reactor utilised in photocatalytic studies

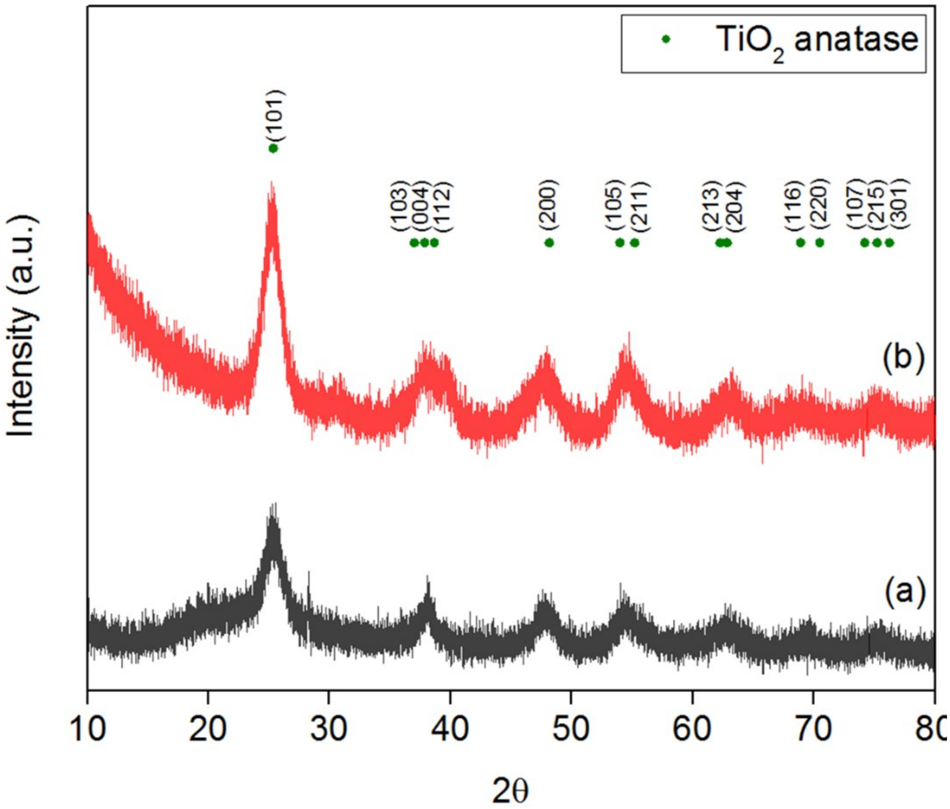

Figure S4. XRD pattern (a) before microwave synthesis of 3.97wt% Pd-TiO_2 powder and (b) after typical 3 minute microwave synthesis

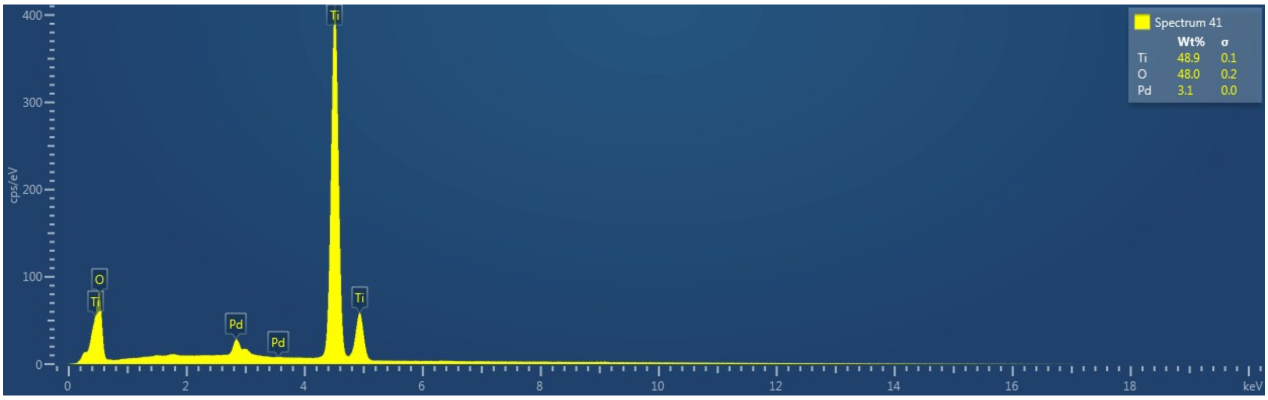

Figure S5. Typical EDX spectrum of a 3.97wt % Pd-TiO_2 powder

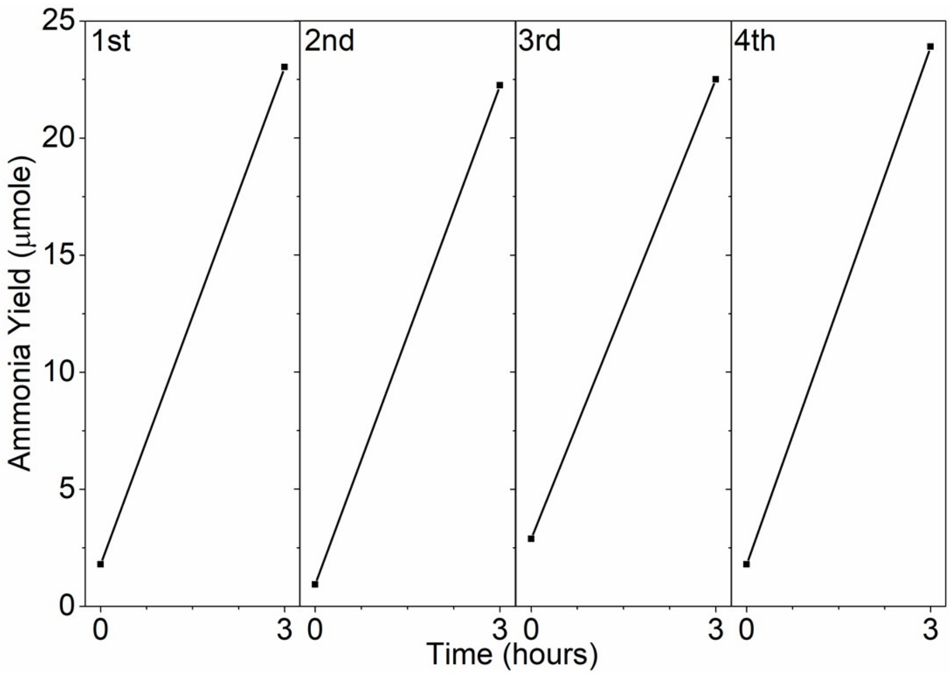

Figure S6. Yield of NH_3 over repeated 3 hour illuminated photocatalytic reactions, after each cycle photocatalyst powder was retrieved, washed and dried before the next reaction. Photocatalytic reaction conditions are

identical as stated in experimental section, however measurements were made before and after reaction instead of every 30 minutes.

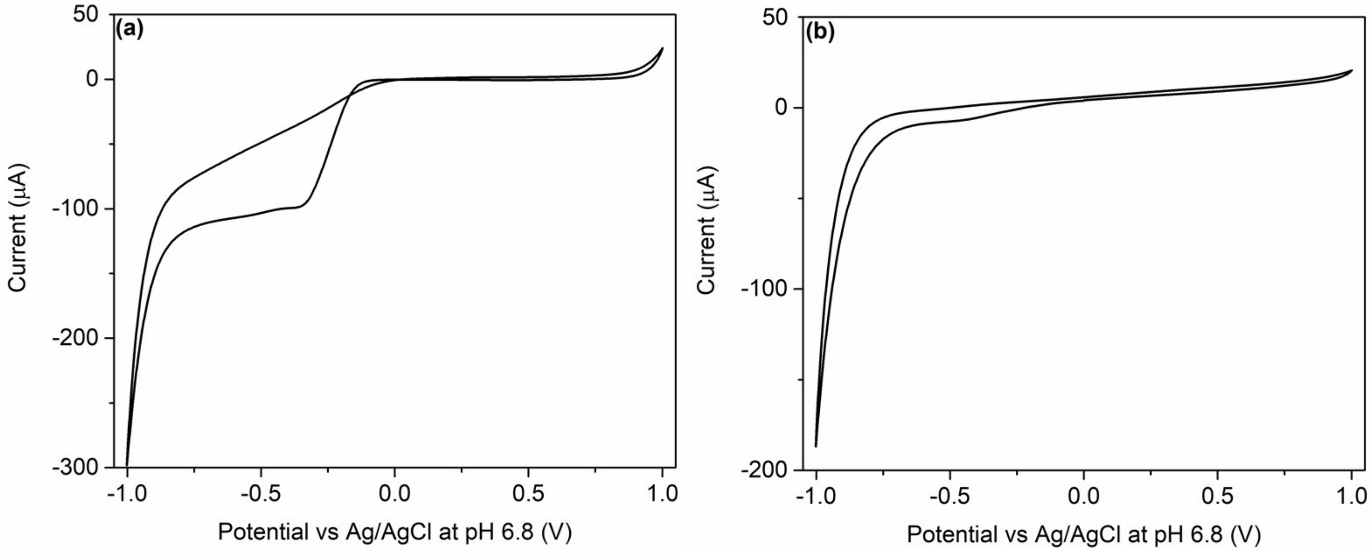

Figure S7. Cyclic voltammograms conducted in the dark on 3.97wt% Pd-TiO2 photoelectrodes across a potential range of +1V to -1V vs Ag/AgCl 3M in 0.2M Na_2SO_4 (pH 6.8) at a scan rate of 20mV/s in (a) Air, and (b) Argon atmospheres

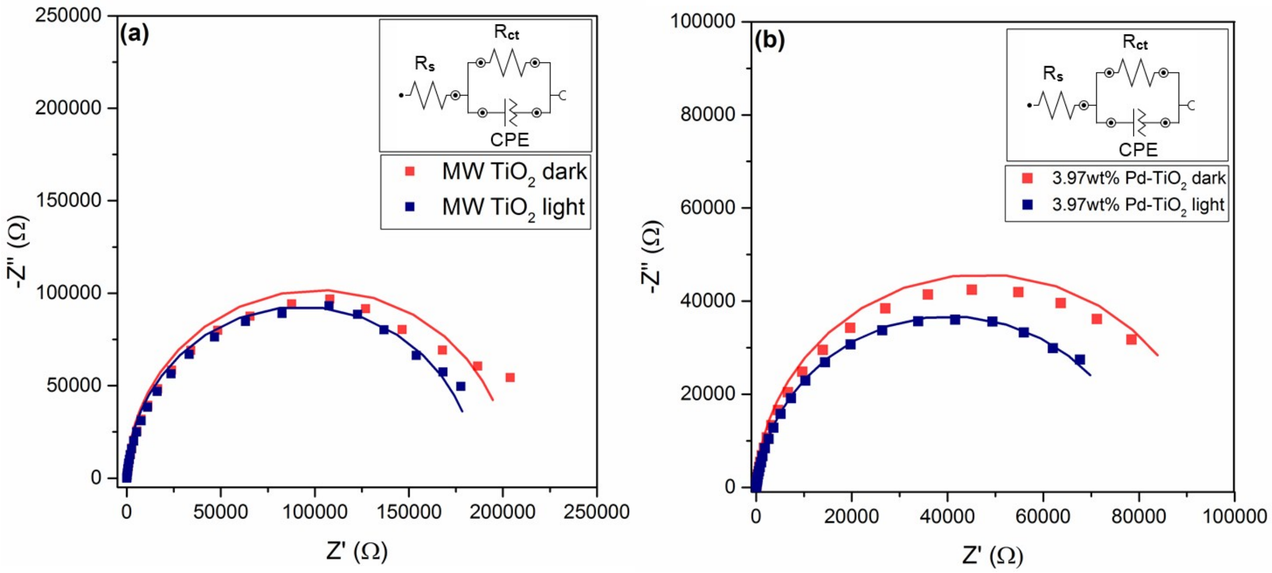

Figure S8. Nyquist plot comparisons between light and dark measurements of (a) MW-TiO_2 and (b) 3.97wt% Pd-TiO_2 photoelectrodes at 0.7V vs Ag/AgCl with a halogen lamp

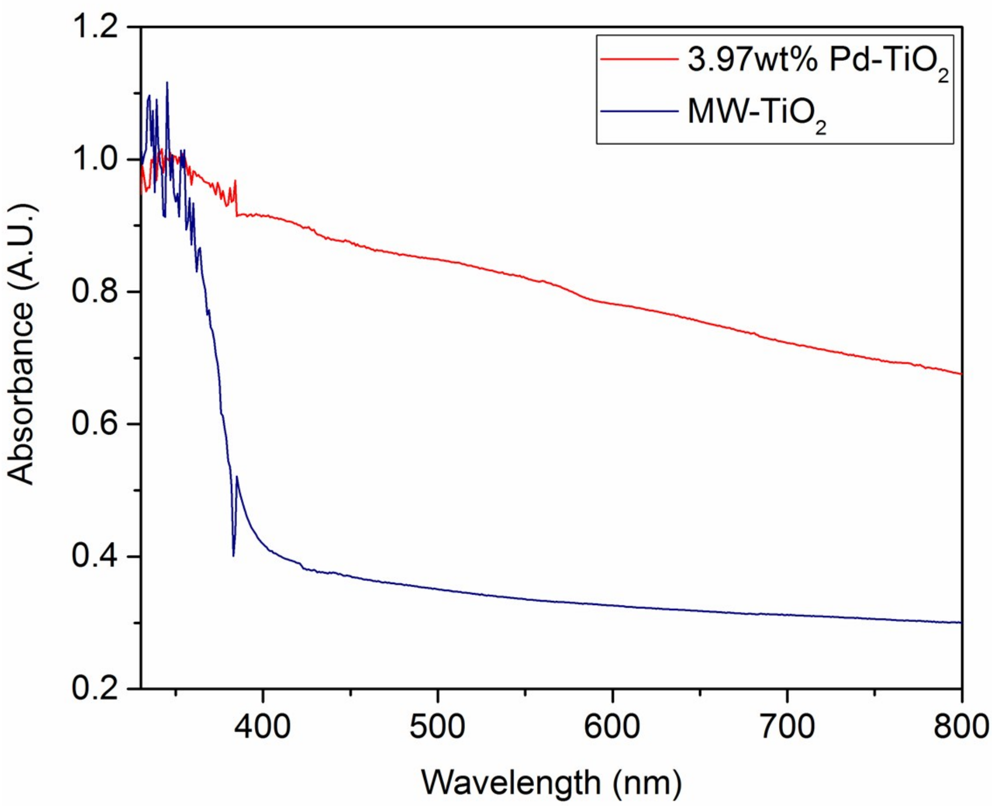

Figure S9. UV/Vis Absorbance spectrum for MW-TiO_2 and Pd-TiO_2 (instrumental noise is seen at lower wavelengths).
